# Contribution of achieving poverty eradication (Sustainable Development Goal 1) to the end tuberculosis strategy in Latin America: a multi-country mathematical modelling analysis

**DOI:** 10.1136/bmjopen-2026-121015

**Published:** 2026-10-27

**Authors:** Ana Paula Santin Bertoni, Felipe Alves Rubio, José Alejandro Ordoñez, Rodrigo Volmir Rezende Anderle, Priscila Fernanda Porto Scaff Gestal, Delia Boccia, Daniella Calvacanti, Davide Rasella

**Affiliations:** 1 Institute of Collective Health, Federal University of Bahia, Salvador, Brazil; 2 São Paulo State University (UNESP), Institute of Chemistry, Araraquara, Brazil; 3 Department of Statistics, Universidad de Concepción, Concepción, Chile; 4 Faculty of Population and Health Policy, London School of Hygiene & Tropical Medicine, London, England, UK; 5 Barcelona Institute for Global Health, Barcelona, Spain

**Keywords:** Public health, Tuberculosis, SOCIAL MEDICINE

## Abstract

**Abstract:**

**Objectives:**

To provide tuberculosis (TB) incidence estimates for different Latin American countries, evaluating the contribution to achieving poverty eradication.

**Design:**

We developed a mathematical model of TB dynamics using ordinary differential equations. Using published data, we estimated TB incidence across various poverty scenarios.

**Setting:**

Latin American countries: Brazil, Colombia, Mexico and Peru.

**Interventions:**

Our analysis included demographics and poverty data across three scenarios: (1) baseline, (2) increasing and (3) decreasing poverty levels.

**Primary and secondary outcome measures:**

We projected trends in new TB notifications from 2020 to 2035 using a TB compartmental model, which incorporates the poverty effects with country-specific data from 2000 to 2019.

**Results:**

The forecasted trends of our poverty eradication scenario by 2030 show reductions in a range of 32.27% (95% CI 36.58% to 25.13%) to 48.56% (95% CI 54.15% to 42.75%) in the TB incidence in comparison with the baseline, with a total number of 516,132 (465,192 to 572,526) avoided TB notifications over the period. The increasing poverty scenario led to an incidence increase ranging from 20.02% (95% CI 5.16% to 35.42%) to 50.20% (95% CI 36.47% to 60.76%) in comparison with the baseline.

**Conclusions:**

Achieving SDG1 of poverty eradication in Latin America by 2030, particularly through the enhancement and expansion of social protection programmes, could significantly contribute to the goals of the End TB Strategy and greatly reduce the tuberculosis burden in low- and middle-income countries (LMICs).

**STRENGTHS AND LIMITATIONS OF THIS STUDY**

- This study applies compartmental mathematical modelling to multi-country data to estimate the impact of poverty reduction on tuberculosis incidence in Latin America, highlighting poverty alleviation’s critical role in combating the TB epidemic.
- The lack of data for specific subgroups in all countries limited the study to the general population by country.
- This study did not consider factors such as the potential introduction of new treatments or diagnostics, due to the lack of data and complexity of the mathematical model, which could affect the projections.

## Introduction

 Tuberculosis (TB) remains a leading cause of death worldwide, claiming nearly 1.25 million lives in 2023.^1^ Low- and middle-income countries (LMICs), in addition to having the highest concentration of poverty, demonstrate the highest burden of TB.^1 2^ The global burden of TB is still largely driven by poverty, income inequalities and food insecurity, as well as inadequate housing and working conditions.^1^ Ending TB requires a comprehensive effort to integrate biomedical advances with interventions addressing the root causes of the epidemic.^2^ This vision aligns with both the Sustainable Development Goals (SDGs) and the WHO’s Multisectoral Accountability Framework for Tuberculosis (TB). It also corresponds with the 2023 Political Declaration from the High-level Meeting of the United Nations General Assembly on the Fight Against Tuberculosis. During this meeting, member states committed to a new TB target, which aims to ensure that by 2027, all individuals affected by TB will have access to a comprehensive package of health and social benefits.^3–5^

Despite advances in the fight against TB in Latin American countries, the challenge of reducing poverty, among others, still hinders progress toward the end of TB in this region.^3^ Recently, Latin America has seen an increase in the incidence of TB along with an increase in poverty and social vulnerability as a result of the COVID-19 pandemic, among other factors.^6^ Poverty is a critical structural and multidimensional social determinant of health (SDH) that can impact other socioeconomic factors,^7^ such as poor housing conditions, lack of environmental sanitation, inadequate nutrition and insufficient personal hygiene and protective measures. These factors, when combined, can increase the probability of TB infection and of TB transitioning from the inactive (latent) TB stage to the development of TB disease (active TB).^8^ Furthermore, lower income levels can harm healthcare-seeking behaviour, leading to delayed TB diagnosis and treatment and lower treatment compliance.^9^ Poverty may also heighten HIV-related vulnerability, as socioeconomically vulnerable populations are more likely to acquire HIV and to face barriers to HIV prevention and care.^10^ HIV infection, in turn, increases the risk of progression from TB infection to active disease.^11^ This may further reinforce the link between poverty and TB. Delayed TB diagnosis and treatment can worsen the outcomes for those who develop active TB, while also increasing the costs for patients, as well as for both public and private health services.^7 9^

Addressing these socioeconomic determinants can significantly reduce TB incidence and improve health outcomes for affected populations, particularly through poverty eradication measures.^2 12^ In this regard, recently, the G20—through the Global Alliance against Hunger and Poverty—has emphasised the importance of social protection programmes to tackle global poverty.^13^ The group advocates for innovative financing mechanisms and international cooperation to support initiatives aimed at this goal, especially in LMIC to address the challenges posed by economic instability and climate change.^13^

This study aims to forecast the impact of alternative scenarios of poverty evolution, including the achievement of Poverty Eradication (SDG1) by 2030^4^ on the TB incidence and the cumulative number of TB notifications by 2035 in the four Latin American countries with the highest TB burden.

## Methods

### Study design and data sources

Our analysis focused on the Latin American (LA) countries with the highest number of TB notifications, based on the most recent statistics from the pre-pandemic year 2019.^1^ These countries are Brazil, Colombia, Mexico and Peru, which together account for approximately 70% of all TB notifications in Latin America (see online supplemental figure S1). The annual data on newly reported TB notifications during the years 2000 to 2019 were collected from the WHO global TB database.^1^

The poverty rate series was extracted for each country from the World Bank,^14^ using the threshold of 6.85 dollars per day based on the 2017 round of the International Comparison Programme (ICP) price data on the International Poverty Line. This rate represents the average of national poverty lines in upper-middle-income countries. In notifications where poverty values were unavailable for certain years, we interpolated the mean value between the nearest preceding and subsequent years to fill in the gaps. Online supplemental table S1 presents the list of LA countries and years for which data were available.

Data on the general mortality rate per 1000 people, life expectancy at birth (total in years) and total population, along with their projections up to the year 2030, were sourced from the World Bank database.^14^

All analysed data were extracted from public repositories, and formal ethics board approval was not required.

### Compartmental model

In our compartmental model, we classified individuals into specific groups based on their health status.^8 15^ To project the number of new active TB notifications from 2020 to 2035, we used a previously described and consolidated compartmental model,^8^ developed specifically to incorporate factors related to poverty and socioeconomic status. This model was composed of four compartments: susceptible (S), primary infection (P), latent infection (L) and active tuberculosis disease (I). The model structure is presented in online supplemental figure S3. Online supplemental table S2 shows the model parameters, their description and the values used in the simulations.

As previously described,^8^ the transmission dynamics of TB are given by the nonlinear ordinary differential equations system (online supplemental equation S1). The range of parameters of the system was determined based on the literature review, while the initial conditions for each country, such as crude death rate per 1000 people, life expectancy at birth and total population, and the projections for 2035, were obtained from the World Bank.^14^ The initial conditions and input variables for each country are detailed in online supplemental table S3, whereas the estimated model parameters are shown in online supplemental table S4. Detailed information about the model transitions diagram, the system of different equations, the parameters used and the initial conditions is available on the online supplemental material pages 4–7.

### Poverty scenarios to 2030

With the objectives of the Sustainable Development Goals (SDGs) of the 2030 Agenda aimed at eradicating poverty in mind, our focus was on modelling the SDG indicator 1.2.1: ‘Proportion of the population living below the national poverty line’.^4^ The poverty headcount ratio at US$6.85 a day (% of the population, 2017 purchasing power parity (PPP)) was employed to estimate the proportion of poverty in each country.

To build the scenarios, we employed an exponential decay function to reach 0% of poverty in 2030, taking into account the initial values in 2019 of each country. Also, we used an exponential growth function to assess a possible scenario of increasing poverty for each country. As a baseline scenario for poverty proportion, we used an Auto-Regressive Integrated Moving Average (ARIMA) model. These scenarios are shown on the online supplemental figure S4.

### Incorporation of poverty into mathematical modelling

As highlighted in the model transitions diagram (online supplemental figure S3), poverty eradication was incorporated into parameters that indicate the transition of individuals from primary infection and from latent infection to active TB notifications. The poverty-related parameter was informed by a published meta-analysis and was based specifically on the subgroup estimate for poor and marginalised populations, rather than on the pooled estimate for all at-risk groups. Bohlbro et al (2021) reported a yield/prevalence ratio of 3.0 (95% CI 2.0 to 3.9) for poor and marginalised populations, compared with 1.4 (95% CI 0.8 to 1.9) in the general population.^16^ These RR were incorporated in both parameters, using a Population Attributable Fraction (PAF)^17^ approximation as previously made.^8^ In summary, the additional risk of TB progression associated with poverty is added to the proportion of the population in poverty (see online supplemental material*,* page 5).

We calibrated the model using the reported incidence of new tuberculosis (TB) notifications for each country and Markov Chain Monte Carlo (MCMC) sampling to obtain posterior estimates, implemented in Python using PyMC v5.7.2. This Bayesian approach allowed us to account for uncertainty in the calibrated parameters throughout the analysis. Rather than basing the projections on a single set of parameter values, we generated predictions across the posterior samples, so that parameter uncertainty was naturally carried forward into the projected TB incidence. In this sense, the procedure provides an assessment analogous to a probabilistic sensitivity analysis, since the variability in the model outputs reflects the joint uncertainty in the estimated parameters. We summarised the resulting posterior distributions using the median and the 2.5th and 97.5th percentiles, corresponding to 95% CI, for both the calibration period (2000 to 2019) and the forecasting period (2020 to 2030). We implemented four chains, each comprising 20 000 draws, and excluded the initial 2000 samples from each chain as burn-in.

### Patient and public involvement

There was no patient or public involvement in this study.

## Results

Among the four countries analysed, there were notable differences in TB and poverty data spanning 2000 to 2019. Table 1 summarises TB-related data and the percentage of the population living on $6.85 or less per day in 2000, 2010, and 2019 for the four analysed countries. In 2019, Brazil, Mexico and Peru reported the highest number of new TB notifications, totalling 155 281 notifications. Peru is the country with the highest TB incidence, achieving 119 notifications per 100 000 inhabitants in 2019, while Mexico is the one with a lower incidence (23 notifications per 100 000). Despite that Peru’s incidence in 2019 was 34.97% lower than in 2000, and 8.19% lower than in 2010. No other country presents a reduction in the time frame 2010–2019. Brazil, Mexico and Colombia exhibited more stable incidence rates by the end of 2019, with rates of 46, 23 and 35 notifications per 100,000, respectively. While poverty rates decreased consistently in the first 2000s decade in all countries (eg, 53.69% in Peru) and smoothed in the second decade (eg, 16.24% in Brazil). All the detailed data can be found on the online supplemental table S4.

**Table 1:** Descriptive analysis of the study indicators and percentage change from 2019 compared with 2000 or 2010 across four Latin American countries

Country	Indicator	2000	2010	2019	2019 Change vs. 2000 baseline
Brazil	New TB notifications	77 899	75 196	85 523	7624
	TB incidence*	51	44	46	−5
	Poverty rate†	50.9%	31.28%	26.2%	−48.53%
	New TB notifications	11 630	11 420	14 292	2662
Colombia	TB incidence*	37	32	35	−2
	Poverty rate†	59.5%	44.6%	34.8%	−41.51%
Mexico	New TB notifications	18 434	20 155	23 702	5268
	TB incidence*	23	22	23	0
	Poverty rate†	48.8%	38.1%	31.8%	−34.84%
Peru	New TB notifications	38 661	31 073	31 764	−6,897
	TB incidence*	183	134	119	−64
	Poverty rate†	62.4%	40.5%	28.9%	−53.69%

* TB incidence (per 100 000 population).

† Population living on US$6.85 or below a day.

TB, tuberculosis.

Table 2 shows the modelled TB incidence for the periods of 2025, 2030, and 2035 on the three scenarios with their respective credible intervals (CI). Without additional interventions, in a hypothetical scenario modelled for poverty eradication by 2030, forecasted trends indicate reductions in TB incidence across the analysed countries. The percentage reduction in 2025 ranged from 18.2% (CI 24.8% to 11.4%) in Peru to 30.2% (CI 37.2% to 23.0%) in Colombia, in 2030 ranged from 26.7% (CI 33.0% to 20.1%) in Peru to 42.9% (CI 48.9% to 36.6%) in Colombia, and 2035 ranged from 32.2% (CI 36.5% to 25.1%) in Brazil to 48.5% (CI 54.1% to 42.7%) in Colombia. Conversely, the increasing poverty scenario would lead to a range of percentage increases, starting in 2025 from 9.4% (CI −1.5% to 20.6%) in Colombia to 12.0% (CI 2.8% to 21.5%) in Peru, going in 2030 from 10.9% (CI −2.7% to 25.1%) in Mexico to 19.9% (CI 8.4% to 31.8%) in Peru, and in 2035 from 20.0% (CI 5.1% to 35.4%) in Colombia to 25.6% (CI 6.9% to 45.2%) in Mexico.

**Table 2:** Estimates of modelled TB incidence (per 100 000 population) with 95% CI by 2025, 2030, and 2035 in four Latin American countries

Country	Year	TB incidence in poverty scenarios			% difference from the baseline scenario	
		Baseline (95% CI)	Increase (95% CI)	Decrease (95% CI)	Increase (95% CI)	Decrease (95% CI)
Brazil	2025	36.9 (34.9 to 40.4)	41.0 (38.8 to 45.1)	29.9 (28.1 to 32.8)	11.3 (5.3 to 22.2)	−18.9 (-23.7 to −11.0)
	2030	35.6 (33.5 to 39.4)	42.0 (39.5 to 46.6)	25.8 (24.2 to 28.4)	17.9 (10.9 to 30.7)	−27.5 (-32.0 to −20.1)
	2035	34.3 (32.2 to 38.2)	42.3 (39.6 to 47.3)	23.2 (21.7 to 25.7)	23.1 (15.3 to 37.7)	−32.2 (-36.5 to −25.1)
Colombia	2025	25.9 (23.4 to 28.5)	28.4 (25.5 to 31.3)	18.1 (16.2 to 19.9)	9.4 (-1.5 to 20.6)	−30.2 (-37.2 to −23.0)
	2030	26.0 (23.1 to 28.9)	29.8 (26.4 to 33.3)	14.8 (13.2 to 16.4)	14.7 (1.8 to 28.0)	−42.9 (-48.9 to −36.6)
	2035	25.7 (22.7 to 28.9)	30.9 (27.1 to 34.9)	13.2 (11.8 to 14.7)	20.0 (5.1 to 35.4)	−48.5 (-54.1 to −42.7)
Mexico	2025	17.7 (15.5 to 19.9)	19.6 (17.2 to 22.1)	14.1 (12.4 to 15.8)	10.9 (-2.7 to 25.1)	−20.3 (-29.7 to −10.7)
	2030	17.2 (14.9 to 19.5)	20.4 (17.6 to 23.4)	12.0 (10.5 to 13.5)	19.0 (2.6 to 35.9)	−29.9 (-38.5 to −21.0)
	2035	16.7 (14.3 to 19.2)	21.0 (17.9 to 24.3)	10.8 (9.4 to 12.2)	25.6 (6.9 to 45.2)	−35.3 (-43.6 to −26.8)
Peru	2025	84.0 (77.2 to 90.9)	94.0 (86.3 to 102.0)	68.6 (63.1 to 74.4)	12.0 (2.8 to 21.5)	−18.2 (-24.8 to −11.4)
	2030	77.7 (70.7 to 85.0)	93.2 (84.2 to 102.5)	56.9 (52.0 to 62.0)	19.9 (8.4 to 31.8)	−26.7 (-33.0 to −20.1)
	2035	73.4 (66.1 to 81.0)	91.8 (81.9 to 102.1)	49.6 (45.1 to 54.3)	25.0 (11.5 to 39.2)	−32.3 (-38.4 to −25.9)

TB, tuberculosis.

Figure 1 illustrates the trends in tuberculosis (TB) incidence over time for each scenario across the modelled countries. The analysis of the calculated TB incidence data from 2000 to 2019 (represented by the black circles in figure 1) indicates an overall decreasing trend. Starting from 2020, the projected TB incidence under various poverty scenarios shows significant variations up to 2035. In the poverty eradication scenario (green line), a sharp decline in TB incidence is anticipated, reflecting the potential benefits of effective poverty alleviation measures. In the baseline scenario (blue line), the projections suggest a more stable trend, with only minor fluctuations over time. Conversely, the increasing poverty scenario (red line) predicts a rise in TB incidence, highlighting the negative impacts that increasing poverty levels may have on public health.

**Figure 1:**
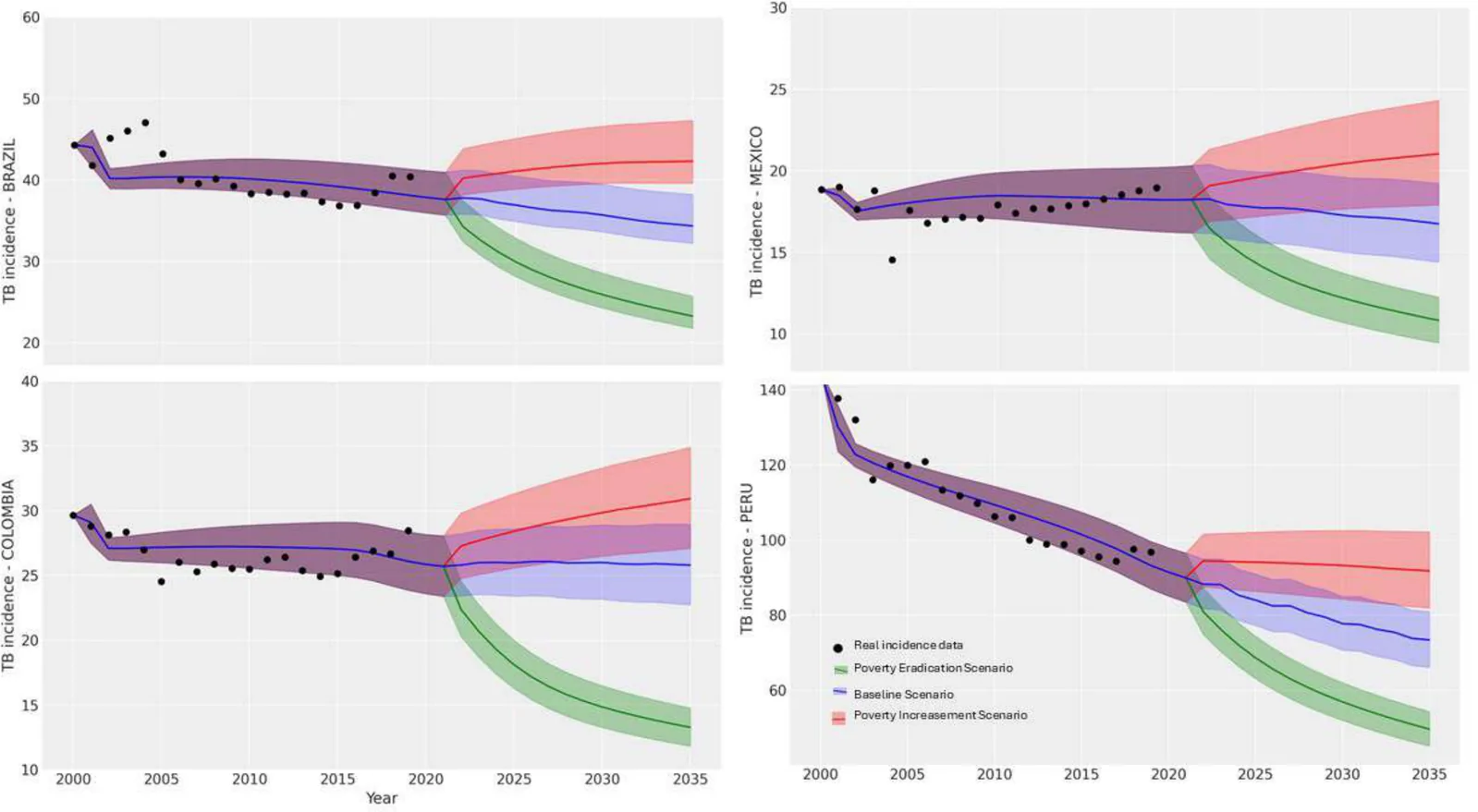
Tuberculosis incidence reported from 2000 to 2019 (depicted by black circles) and modelled TB incidence (represented by solid lines) covering from 2020 to 2035, under different poverty scenarios, such as increasing (red), mitigating poverty (green), and the baseline scenario (blue). TB, tuberculosis.

The cumulative number of new TB notifications from 2020 to 2035 is illustrated in figure 2. Our simulations indicate that if the 2030 poverty eradication goal is achieved, it may be possible to prevent 516 132 new notifications of active TB by 2035 across the four analysed Latin American countries. Conversely, if poverty increases, we could see an additional 318 426 new notifications of active TB by 2035 in these countries. The difference between these two extreme scenarios represents a total of 834 558 cumulative TB notifications over this period. As expected, Brazil concentrates most of the accumulated notifications, followed by Peru, Mexico and Colombia.

**Figure 2:**
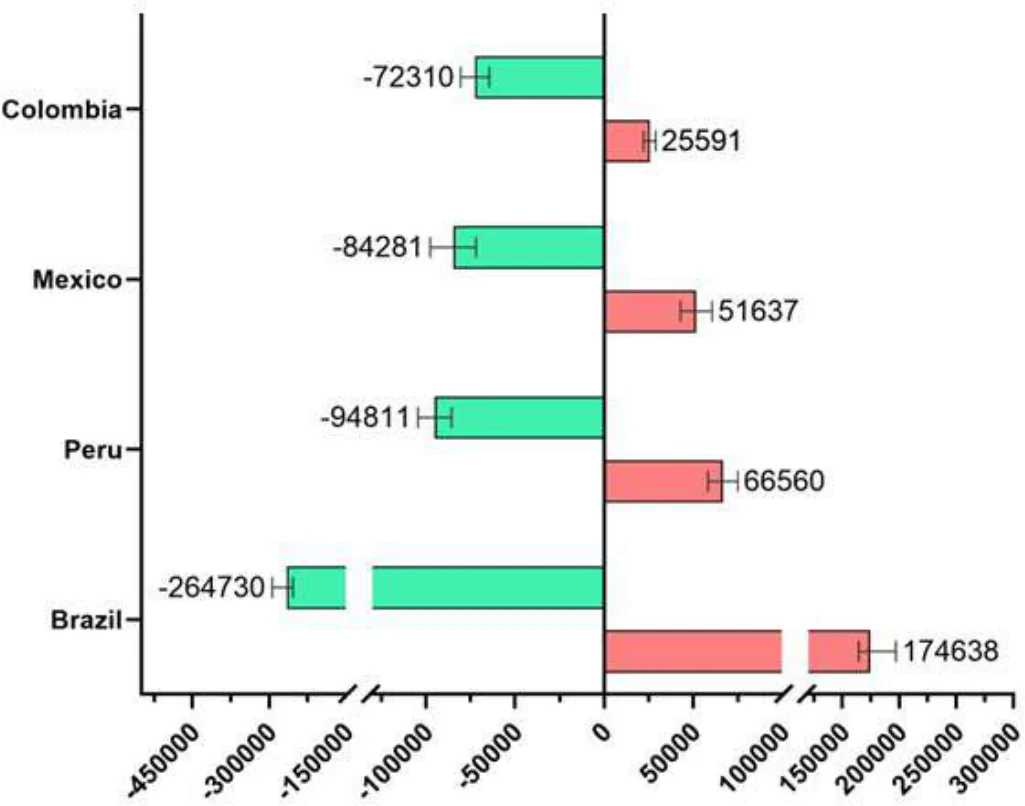
Cumulative modelled notifications of tuberculosis in Latin American countries, under hypothetical increasing (red) and decreasing (green) poverty scenarios compared with baseline scenarios, 2020–2035.

Table 3 provides a detailed overview of changes in TB incidence across various scenarios for each country. It highlights how these changes align with the targets set by the End TB Strategy – a 90% reduction in TB incidence by 2035.^3^ In the scenario focused on poverty eradication, Peru shows a significant decrease in TB incidence, with a reduction of 48.87% by 2035 compared with the baseline year of 2015. Colombia also sees a notable decrease, with a 47.32% reduction in TB incidence by 2035 against the same reference year. Mexico and Brazil show lesser reductions at 39.82% and 36.88%, respectively. By consequence, the verified reductions indicate that the impacts in Peru and Colombia could exceed 50% of the 90% reduction target set by the End TB Strategy, while the changes in Mexico and Brazil could represent more than 40% of this target.

**Table 3:** Impact of the modelled scenarios on TB incidence by 2035 in four countries of the Latin America region

	Poverty scenarios	Brazil	Colombia	Mexico	Peru
Percentual difference in 2035 compared with the modelled baseline	Increase (%)	23.18	20.02	25.63	25.07
	Decrease (%)	−32.27	−48.56	−35.36	−32.33
Change from 2015 to 2035 according to the scenario	Baseline (%)	−6.81	2.42	−6.90	−24.44
	Increase (%)	14.79	22.92	16.96	−5.50
	Decrease (%)	−36.88	−47.32	−39.82	−48.87

2015 has been chosen due to the WHO End TB Strategy target of 90% reduction by 2035, related to 2015.

## Discussion

Our study shows for the first time that the achievement of Poverty Eradication SDG1 can consistently and significantly contribute to ending TB in four Latin American countries. Through a gradual reduction of poverty since 2020 and its complete elimination by 2030 in countries such as Brazil, Colombia, Mexico and Peru, our model estimates a potential decrease of 32.3% to 48.6% in new notifications of active TB by 2035. According to our poverty eradication scenario, it could be possible to avert more than half a million new notifications of active TB by 2035 (figure 2).

The four countries analysed in our study accounted for over 70% of the total TB notifications in Latin America in 2019 (online supplemental figure S1). More recent WHO estimates indicate that TB incidence increased again in Brazil and Peru in 2022.¹ These post-2019 estimates are provided only to contextualise recent epidemiological trends and were not included in the model calibration. Brazil and Peru are among the WHO high-TB burden countries.^6^ Even more notably, Mexico and Colombia also reported an increase in TB incidence not observed since the year 2000.^1^ Other Latin American countries with smaller populations have also faced challenges in addressing the decrease in new notifications and TB-related deaths.^6^ During 2020, reported TB notifications declined owing to pandemic-related disruptions in diagnostic services, which did not necessarily reflect a true decline in TB incidence. The subsequent increase in reported TB cases may partly reflect the recovery of diagnostic and notification services following the COVID-19 pandemic. On the other hand, the end of the pandemic also brought with it an increase in poverty, which, together with the economic and social crisis, meant an increase in the homeless and incarcerated population in the region of the Americas, factors related to the increase in TB disease and transmission.^6^

Concerning the WHO’s targets for the End TB Strategy for 2035 (online supplemental figure S2),^3^ our estimates suggest that achieving poverty eradication by 2030 could contribute to more than 50% of the WHO’s 90% TB incidence reduction target in Peru and Colombia, and more than 40% of this target in Brazil and Mexico. Our results align with previous findings that suggest eradicating extreme poverty could result in a 33.4% (95% CI 15.5 to 44.5) reduction in global TB incidence by 2035.^12^ However, our study demonstrates even stronger effects due to the application of a more advanced approach in integrating poverty into the infectious disease dynamics of TB, along with the use of innovative compartmental modelling techniques.

While the goal of eradicating poverty by 2030 (SDG1) may seem increasingly unlikely, the findings of this study underscore the importance of prioritising poverty alleviation policies as a cornerstone for the success of global strategies like the End TB initiative. Decisions such as increasing military budgets,^18^ while critical sectors like health and research face significant cuts,^19 20^ exemplify how political choices can divert resources away from areas that promote equity and social well-being. This study reinforces that, even in uncertain times, investing in social protection and poverty reduction is essential to achieving global goals and improving public health.

In addition to the healthcare system disruptions, the COVID-19 pandemic and other recent crises have led to an increase in poverty rates, which has only worsened existing inequalities.^21^ Thus, given that poverty is still the key driver of TB globally,^21 22^ social protection measures addressing poverty and inequalities should be critical or essential parts of countries’ TB response. This does not fall under the responsibility of National TB Programmes (NTPs); however, NTPs can play an important role in promoting the implementation of TB-sensitive social protection programmes and ensuring that ending TB becomes part of the development agenda.^7^ Furthermore, NTPs can contribute to breaking the underlying mechanism linking TB and poverty by addressing some of the mediators along the pathway: for example, NTPs can advocate for the improvement of housing conditions to reduce exposure to crowding and poor ventilation. They can also claim that the transition from burning fuels like wood, charcoal, coal and kerosene to cleaner alternatives can decrease indoor air pollution, another contributing factor to TB. Additionally, NTPs can act on behaviours such as smoking and alcoholism, which increase TB risk. These interventions can also mitigate other diseases that contribute to TB incidence and mortality, such as HIV and AIDS, which are higher among socially vulnerable populations.^10 23–25^

Finally, NTPs can strengthen the implementation of TB-specific social protection programmes under their responsibility to mitigate the financial burden of TB in TB-affected households and reduce the extent of impoverishment resulting from TB.^7^

The G20 has increasingly recognised the vital role of social protection programmes in addressing global poverty, especially as nations face economic instability, climate change and the challenges of post-pandemic recovery.^13^ As a forum comprising the world’s largest economies, the G20 has emphasised the importance of innovative financing mechanisms and international cooperation to sustain and expand social protection systems.^13^ Such a global focus reinforces the efforts to achieve the UN Sustainable Development Goals, particularly Poverty Eradication (SDG1).^4^

Considered a human right, social protection is an intervention for providing resources, most often in cash, that is essential for reducing social inequalities. Through social protection, beneficiaries achieve poverty alleviation, income stability and access to healthcare services.^26^ Conditional cash transfer (CCT) programmes act as a fundamental social protection strategy and in addition to reducing poverty, they promote social rights and access to primary healthcare services^27^ and the mitigation of childhood mortality^23^ and morbidity and mortality from various diseases such as cardiovascular problems,^25^ mental illnesses^24^ and infectious diseases such as HIV/AIDS^10^ and tuberculosis.^28^ The positive effects of CCT have been demonstrated in Peru through the CRESIPT socioeconomic support intervention, with reductions of catastrophic costs related to TB illness and enhancement of TB prevention and treatment,^29^ as well as in Brazil through the Bolsa Família Programme for several TB outcomes, like reducing the incidence, mortality, dropout and increasing the cure rate of TB.^30 31^ A recent study highlights the association between CCT and significant reductions in TB incidence and mortality, with even greater reductions observed in more vulnerable subpopulations.^28^

Social protection is one of the key components for achieving the TB elimination targets^1 3 28^ as it promotes the alleviation of one of the main factors associated with TB, poverty.^30^ Beyond this main mechanism, access to CCT benefits makes it possible to reduce financial costs related to TB diagnosis and treatment^1 31 32^ and promote improvements in housing and ventilation conditions that reduce intra-household transmission of the disease.^31^ CCT also promotes access to food for its beneficiaries,^31^ thus combating food insecurity, an important social determinant for TB and failure in treatment.^12^ In addition to access to food, undernourishment rates can also be reduced in the presence of high social protection coverage, even in economic crisis scenarios^21^

Access to social protection programmes also encourages health-seeking behaviours, which are important for early diagnosis, adherence to preventive treatment for latent TB and completion of treatment for active TB.^12^ Generally, the conditionalities of CCTs are related to access to education and health services, especially maternal and child care, such as prenatal monitoring for pregnant women, vaccination and nutritional status for young children and school attendance.^26 27^ Although conditionalities are not directly related to TB care,^31^ they act as stimulating interventions for access to public services, covering the entire family of beneficiaries.^27^ When accessing health services, CCT beneficiaries receive information about the symptoms, diagnosis and treatment of TB, which can impact improvements in the prevention and care of the disease.^30^

Our model has limitations. First, we did not consider factors such as the potential introduction of new treatments or diagnostics, which could affect the projections. The primary aim of our study was not to explicitly forecast the future burden of TB over the next decade. Instead, we sought to understand the disparities in TB projections related to the poverty eradication goal (SDG 1) and the End TB Target strategy across four Latin American countries. We assumed that the same factors and historical events have similarly influenced the scenarios and trends of the disease across these nations, ensuring that our comparisons remain robust in magnitude. In this context, we intentionally chose not to delve into the unique characteristics and specificities of each country, as our objective was to create a broader overview of the potential impact of poverty reduction on the burden of TB in this region. Moreover, we didn’t explicitly model for COVID-19 due to the lack of quality data and the complexity of representing the healthcare disruption related to TB during this extremely challenging period. However, we assume that for the majority of analysed countries, the situation has returned to normal in recent years and is not affecting our long-term estimates. Second, with our approach, we were only able to differentiate individuals between poor and not poor, without representing in detail more granular population characteristics. Even models that stratified compartmental models, which try to separate individuals into different groups, face challenges when it comes to representing this kind of variety.^33^ The only possibility to correctly account for the population’s heterogeneity would be through individual-based models, which require parameters that are extremely difficult to quantify and calibrate.^34^ We implement a reductionist perspective,^33^ by addressing a multidimensional factor (poverty) in the disease dynamic, based on an innovative and recently used approach for representing SDH in infectious diseases.^8^ If anything, due to these limitations mentioned earlier, our results may underestimate the true impact of poverty on TB incidence. Therefore, we should consider our findings as a lower estimate of the potential benefits of poverty eradication in these countries.

In conclusion, achieving the poverty eradication SDG1 in Latin America by 2030—primarily through the strengthening and expansion of social protection policies—could substantially contribute to ending TB in the Latin American region.

## Supplementary material

10.1136/bmjopen-2026-121015 online supplemental file 1

## Data Availability

Data are available in a public, open access repository. The TB and poverty rate datasets generated and analysed during the current study are available in the WHO global TB database, https://www.who.int/teams/global-tuberculosis-programme/tb-reports/global-tuberculosis-report-2024 and the World Bank Open Data https://data.worldbank.org., respectively.
