# Supplementary material for: Contribution of achieving poverty eradication (Sustainable Development Goal 1) to the end tuberculosis strategy in Latin America: a multi-country mathematical modelling analysis
Source: BMJ Open. 2026 Oct 27;16(10):e121015. doi: 10.1136/bmjopen-2026-121015 (PMC13723821; doi:10.1136/bmjopen-2026-121015)
Supplement: Supplementary file 1 [file bmjopen-16-10-s001.pdf]

Supplementary Material

Contribution of achieving poverty eradication (Sustainable Development Goal 1) to the end tuberculosis strategy in Latin America: a multi-country mathematical modelling analysis

Ana Paula Santin Bertoni et al.

Summary

Tuberculosis in Latin America 2

Model Description and Parameters 4

Population Attributable Fraction (PAF) approach 5

Poverty Scenarios 8

Complementary Results 9

References 10

List of Tables

Table S1: Historical series of TB incidence rates (per 100,000 people), total population, and percentage of population living below of the International Poverty Line of $6.85 per day across Latin American countries from 2000 to 2019. 2

Table S2: Description and values for the model parameters. 5

Table S3: Initial conditions and input variables values for each country in 2000 and between 2000-2035.6

Table S4: Estimates of non-cumulative modeled new TB cases in five Latin American countries by 2025, 2030, and 2035. 9

List of Figures

Figure S1: Percentage of new tuberculosis cases reported in Latin America by country, 2019. 3

Figure S2: The areas shaded in light pink represent the historical data on TB incidence, while the green-colored sections depict the projected curve aiming to achieve the targets of the End TB Strategy for 2035, including milestones for each respective country. 3

Figure S3: Schematic diagram of compartmental transmission dynamics TB model. 4

Figure S4: Historical and predictive scenarios of poverty rates, using a poverty line of $6.85/day for the analyzed countries, 2000-2035 8

Tuberculosis in Latin America

Table S1: Historical series of TB incidence rates (per 100,000 people)[1], total population, and percentage of population living below of the International Poverty Line of $6.85 per day across Latin American countries from 2000 to 2019.[2]

		2000	2001	2002	2003	2004	2005	2006	2007	2008	2009	2010	2011	2012	2013	2014	2015	2016	2017	2018	2019
**Brazil**	**TB Incidence**	51	48	52	53	54	50	46	46	46	45	44	44	44	44	43	43	43	44	46	46
	**Population**	175873720	178211881	180476685	182629278	184722043	186797334	188820682	190779453	192672317	194517549	196353492	198185302	199977707	201721767	203459650	205188205	206859578	208504960	210166592	211782878
	**Poverty line at $6.85**	50,90%	48,00%	47,30%	48,70%	47,30%	45,00%	41,40%	39,00%	35,80%	33,80%	31,85%	29,90%	28,20%	25,70%	24,30%	25,30%	27,00%	27,10%	26,70%	26,20%
**Colombia**	**TB Incidence**	37	36	35	35	33	30	32	31	32	32	32	33	33	32	31	31	33	33	33	35
	**Population**	39215135	39837875	40454050	41057687	41648268	42220940	42772910	43306582	43815313	44313917	44816108	45308899	45782417	46237930	46677947	47119728	47625955	48351671	49276961	50187406
	**Poverty line at $6.85**	59,50%	67,00%	57,70%	58,00%	57,30%	53,50%			48,50%	47,30%	44,60%	41,60%	39,80%	37,60%	35,40%	34,60%	34,30%	33,70%	33,90%	34,80%
**Mexico**	**TB Incidence**	23	24	22	23	18	22	22	21	21	21	22	21	22	22	22	22	22	23	23	23
	**Population**	97873442	99394288	100917081	102429341	103945813	105424042	106886790	108302973	109684489	111049428	112532401	114150481	115755909	117290686	118755887	120149897	121519221	122839258	124013861	125085311
	**Poverty line at $6.85**	48,20%		45,30%		42,10%	40,90%	37,20%		37,80%		38,10%		35,40%		38,30%		33,60%		31,10%	
**Peru**	**TB Incidence**	183	173	166	146	151	151	153	143	141	138	134	133	126	125	125	122	120	119	119	119
	**Population**	26654439	27014909	27334503	27623341	27893911	28147267	28381078	28600387	28806185	29009326	29229572	29477721	29749589	30038809	30353951	30711863	31132779	31605486	32203944	32824861
	**Poverty line at $6.85**	62,40%	63,90%	60,20%	59,40%	59,80%	61,50%	55,90%	50,30%	46,40%	44,60%	40,50%	38,10%	35,30%	34,50%	33,40%	32,70%	31,80%	32,60%	30,40%	28,90%

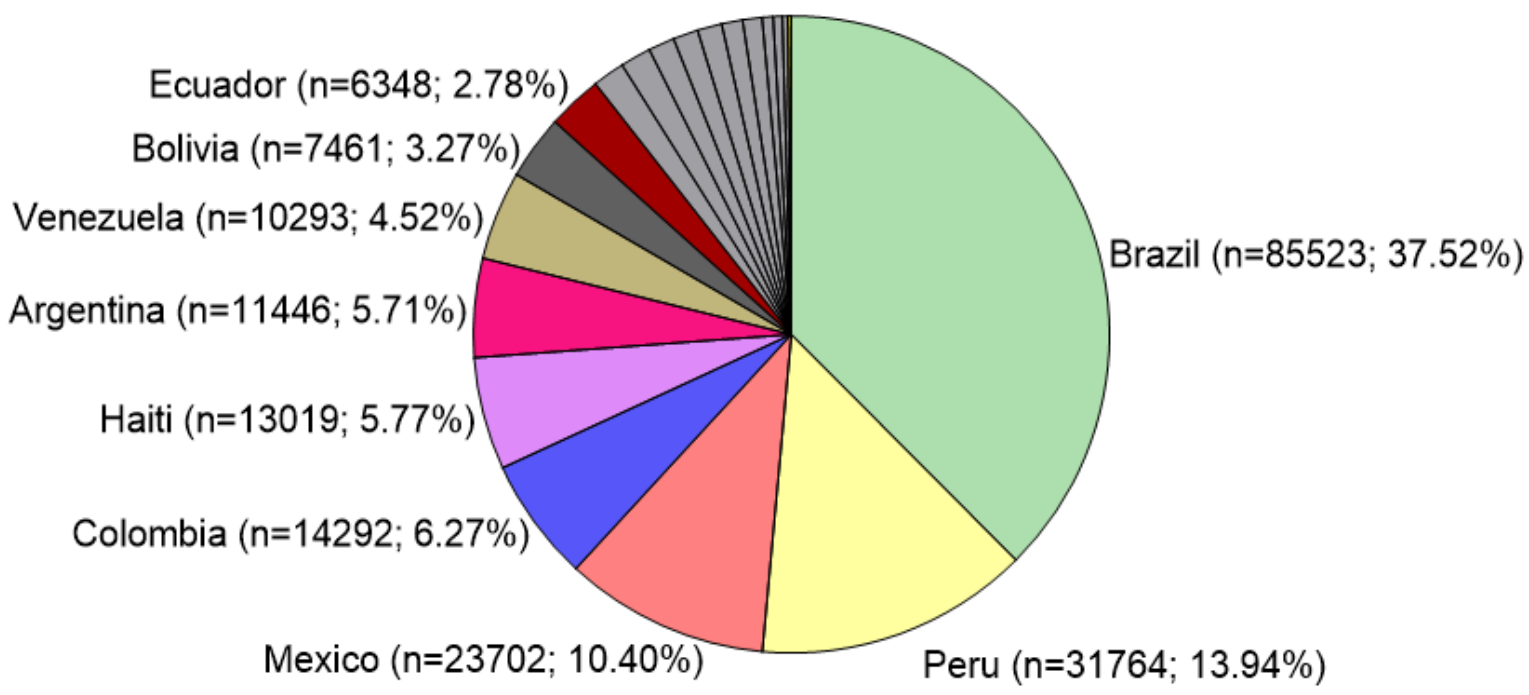

Figure S1: Percentage of new tuberculosis cases reported in Latin America by country, 2019.

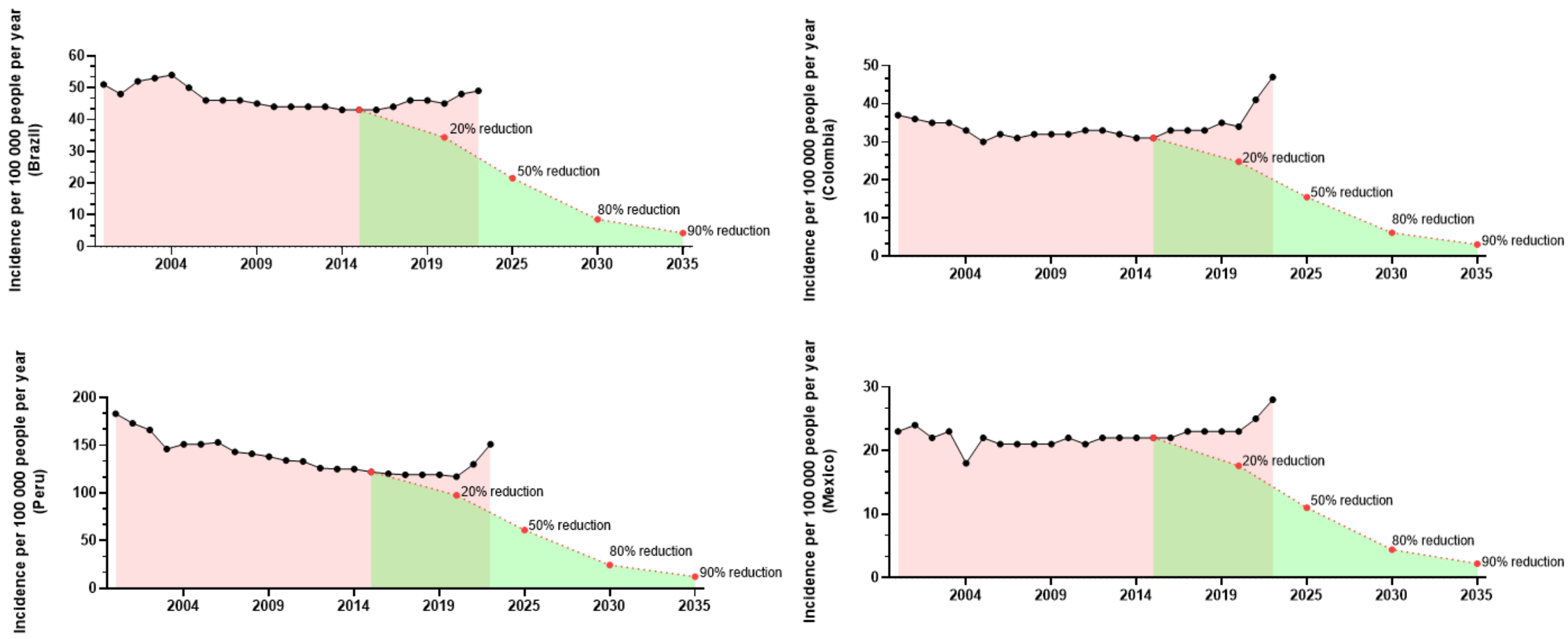

Figure S2: The areas shaded in light pink represent the historical data on TB incidence, while the green-colored sections depict the projected curve aiming to achieve the targets of the End TB Strategy for 2035, including milestones for each respective country.

Model Description and Parameters

The tuberculosis model was based on the work of Gomes et al. (2019), which presented a mathematical model composed of four nonlinear ordinary differential equations, which represents susceptible (S), primary infection (P), latent infection (L), and active tuberculosis disease (I). We assume that only susceptible individuals have a birth rate Λ but all compartments have a natural death rate μ. Contact with an infectious individual (I), represented by the parameter β_T, results in a susceptible individual becoming infected with a primary infection (P). After a period ν^{-1} individuals in the compartment P, a fraction ϕ acquire active tuberculosis, while a fraction (1−ϕ) move to the latent compartment (L). Individuals with active tuberculosis have an additional mortality rate μ_T due to the tuberculosis disease. We consider that through treatment, represented by the per-capita rate π , a fraction θ completely eliminate the bacteria, which makes this individual return to the susceptible compartment. On the other hand, a fraction (1−θ) cannot eliminate the bacteria and the infection becomes latent (L). These individuals in the latent compartment can become infectious when in contact with infectious individuals I or through reactivation and will return to the compartment I at a per-capita rate ω. We also assume that latent individuals can return to the susceptible compartment at a per-capita rate. Figure S3 shows the all compartments considered in the TB transmission dynamics.

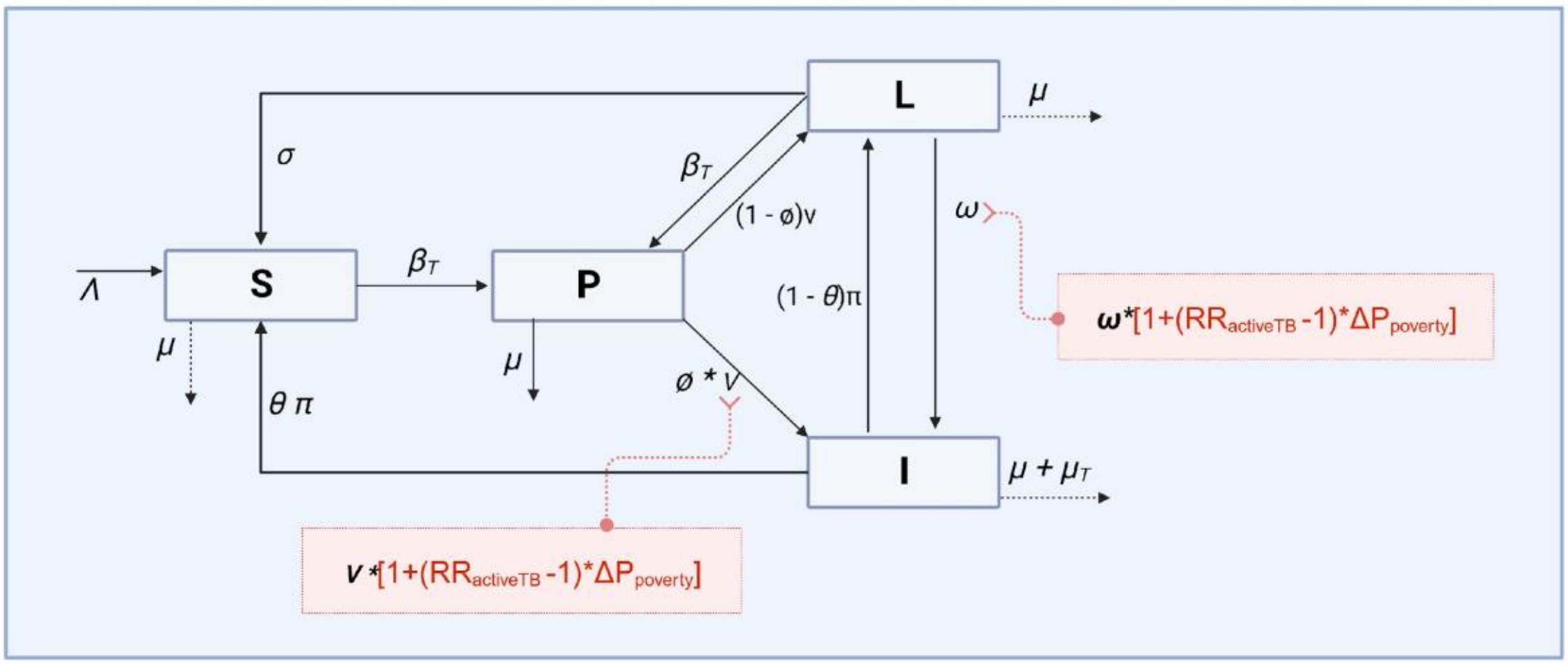

Figure S3: Diagram of the compartmental TB transmission dynamics with (S) susceptible, (P) primary infection, (L) latent infection, (I) active tuberculosis disease.

Equation S1: Nonlinear system of ordinary differential equations.

$$\frac{dS}{dt} = \Lambda - \mu S - \beta_T \frac{IS}{N} + \theta \pi I + \sigma L$$
$$\frac{dP}{dt} = \beta_T \frac{(IS + LI)}{N} - (\nu + \mu)P$$
$$\frac{dI}{dt} = \phi \nu P + \omega L - (\mu_T + \mu + \pi)I$$
$$\frac{dL}{dt} = (1 - \theta)\pi I + (1 - \phi)\nu P - \beta_T \frac{LI}{N} - (\omega + \mu + \sigma)L$$

Table S2: Description and values for the model parameters.

Parameter	Description and source	Value [interval]	Reference
θ	Proportion clearing latent infection upon treatment	[0.704, 0.751]	Derived from [10,11]
ϕ	Proportion progressing from primary infection to active disease	[0.05, 0.15]	[3,4]
π	Rate of successful treatment	[0.63,2]	[4,5]
σ	Progression rate to S from L	0.01	Assumed
μ_T	TB-related death rate	[0.03, 0.08]	[4]
β_T	Progression S to P and Progression L to P	[0,10]	Assumed
ω	Rate of reactivation of latent infection	[0.0013, 0.0084]	[6,7]
ν	Rate progression from primary infection	[2,4]	[8]

Population Attributable Fraction (PAF) approach

From the Population Attributable Fraction (PAF) definition[9], we have

$$PAF = \frac{I_e - I_0}{I_e} \qquad (1)$$

where I_e and I_0 stand for the incidence in exposed subjects and not-exposed subjects, respectively. Also, we can express the equation in terms of the risk ratio (RR), given by

$$PAF = \frac{\rho_e(RR-1)}{1+\rho_e(RR-1)} \qquad (2)$$

where RR represents the risk ratio and ρ_e is the prevalence in exposed individuals.

From the equations (1) and (2), we can rewrite the PAF equation as

$$I_e = \frac{I_0}{1-PAF}$$

$$I_e = \frac{I_0}{1-\frac{\rho_e(RR-1)}{1+\rho_e(RR-1)}}$$

$$I_e = \frac{I_0}{\frac{1+\rho_e(RR-1)-\rho_e(RR-1)}{1+\rho_e(RR-1)}}$$

$$I_e = I_0[1+\rho_e(RR-1)] \qquad (3)$$

Based on the equation (3) we approximated the risk associated with poverty into the parameters of the model such as ν and ω, as follows

$$\omega(t) = \omega_0[1+(RR-1)p(t)]$$

$$\nu(t) = \nu_0[1+(RR-1)p(t)]$$

where $p(t)$ is the annual poverty rate.

Table S3: Initial conditions and input variables values for each country in 2000 and between 2000-2035.

	Brazil	Colombia	Ecuador	Mexico	Peru
Susceptible (S0) [a]	121498031.1	34769665.38	12499705.34	88019898.7	18501714.1
Primary infection (P0) [b]	290000	40000	17500	79000	110000
Active TB (I0) (Cases by 2000 + (% of under notification)) [c]	77899 (+10%)	11630 (+15%)	6908 (+20%)	18434 (+15%)	38661 (+20%)
Latent infection (T0) [d]	$54*10^6$	4392095.12	12499705.34	9787344.20	9787344.20
Birth rate (2000-2035) [e]	3018495.785 30696	760946.2367 4096	311613.6225 938	2163015.70038792	601257.71320484
µ - Natural mortality rate (2000-2035) [f]	1/73.43	1/74.95	1/75.64	1/74.33	1/74.28

a – **Susceptible (S0):** For each country, the initial value of the susceptible individuals was assumed as the difference between the total population and the sum of the other compartments, that is,

S0=N-P0-I0-L0

where N is the total population in 2000.

b – Primary infection (P0): The initial value for the Primary infection was calibrated for each country based on the WHO Global Tuberculosis Programme database variable c_newinc, for the year 2000, representing the total number of notified TB cases, including new cases, relapse cases, and cases with unknown previous treatment history. The under-notification percentage was chosen based on the best model fit within a plausible range of 0% to 39.2%, informed by the WHO regional profile for the Americas, which estimated that 321,000 of 819,000 TB cases were not notified or not diagnosed in 2019 (39.2%). Source: https://www.who.int/teams/global-tuberculosis-programme/data#gtbr, and https://iris.who.int/server/api/core/bitstreams/75cba848-3a89-4e05-a736-afb69d0bece5/content

c – **Active TB (I0):** based on the number of new TB notifications reported in 2000 plus a country-specific percentage of under-notification, used to approximate potentially unreported cases and improve model initialization. Source: WHO Global Tuberculosis Programme database, *"Case notifications"*.
Source: https://www.who.int/teams/global-tuberculosis-programme/data#gtbr

d – **Latent infection (L0):** approximated as 30% of the susceptible population, based on the assumption that about 30% of individuals exposed to *Mycobacterium tuberculosis* become infected.
Source:https://bvsms.saude.gov.br/bvs/publicacoes/manual_recomendacoes_controle_tuberculose_brasil_2_ed.pdf

e - Birth rate (): obtained from the World Bank Population Estimates and Projections database. For each country, the value used in the model corresponds to the average across the years included in the simulations (2000 to 2035). Source: https://databank.worldbank.org/source/population-estimates-and-projections

f – Natural mortality rate (): obtained from the World Bank Population Estimates and Projections database. For each country, the value used in the model is the average value of the inverse of the life expectancy across the years included in the simulations (2000 to 2035). Source: https://databank.worldbank.org/source/population-estimates-and-projections

Table S4: Estimated parameters for the TB model.

**Parameter**	**Country**				
	**Brazil**	**Colombia**	**Ecuador**	**Mexico**	**Peru**
θ	0.730	0.730	0.730	0.730	0.730
ϕ	0.1205	0.1205	0.1205	0.1205	0.1205
π	0.66	0.66	0.66	0.66	0.66
σ	0.01	0.01	0.01	0.01	0.01
μ_{TB}	0.035	0.035	0.035	0.035	0.035
β	5.510	5.550	5.510	5.550	5.550
ω	0.0001350	0.000240	0.000830	0.00020	0.000230
ν	2.5	2.6	3.5	3.8	2.8

Poverty Scenarios

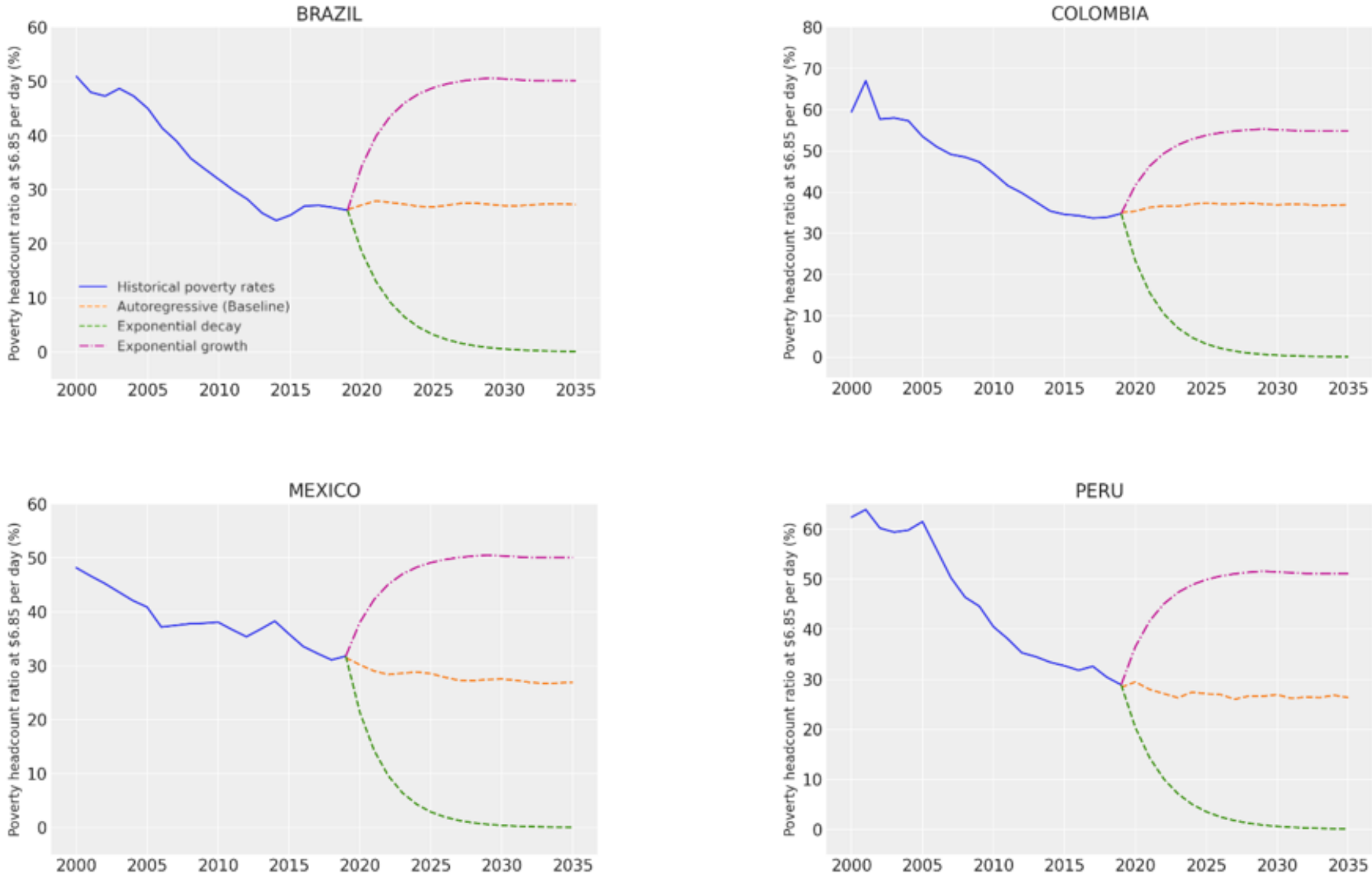

Figure S4: Historical and predictive scenarios of poverty rates, using a poverty line of $6.85/day for the analyzed countries, 2000-2035

Complementary Results

Table S4: Estimates of non-cumulative modeled new TB cases in five Latin American countries by 2025, 2030, and 2035.

Country	Indicator	Absolute number of active TB with 95% credible intervals from different poverty scenarios		
		**Baseline**	**Increase**	**Decrease**
		**n (95% CI)**	**n (95% CI)**	**n (95% CI)**
Brazil	TB cases in 2025	80740 (76363;88545)	89867 (85077;98682)	65444 (61523;71779)
	TB cases in 2030	79826 (75193;88228)	94116 (88575;104391)	57858 (54216;63752)
	TB cases in 2035	78199 (73384;87029)	96309 (90185;107730)	52959 (49586;58546)
Colombia	TB cases in 2025	13660 (12312;15036)	14947 (13451;16473)	9525 (8568;10514)
	TB cases in 2030	14075 (12542;15650)	16155 (14335;18025)	8034 (7182;8919)
	TB cases in 2035	14286 (12595;16036)	17140 (15018;19339)	7348 (6548;8180)
Mexico	TB cases in 2025	23077 (20283;25952)	25607 (22441;28869)	18376 (16206;20594)
	TB cases in 2030	23152 (20113;26304)	27549 (23773;31479)	16225 (14226;18275)
	TB cases in 2035	23135 (19879;26537)	29063 (24740;33612)	14950 (13039;16919)
Peru	TB cases in 2025	29417 (27052;31852)	32947 (30243;35748)	24048 (22111;26052)
	TB cases in 2030	28532 (25960;31201)	34211 (30931;37631)	20901 (19098;22772)
	TB cases in 2035	28109 (25320;31023)	35153 (31363;39131)	19021 (17299;20809)

References

1. *Global Tuberculosis Report 2023*. (World Health Organization, Geneva, 2023).
2. World Bank Open Data. https://data.worldbank.org.
3. Pai, M. & Behr, M. Latent *Mycobacterium tuberculosis* Infection and Interferon-Gamma Release Assays. *Microbiol Spectr* **4**, 4.5.24 (2016).
4. Rubio, F. *et al. Impact of the COVID19-Related Economic Crisis and Mitigation Effects of Social Protection on HIV/AIDS and Tuberculosis: An Integrated Economic, Mathematical and Epidemiological Modelling Study*. https://www.researchsquare.com/article/rs-1316131/v1 (2022) doi:10.21203/rs.3.rs-1316131/v1.
5. Steffen, R. E., Pinto, M., Kritski, A. & Trajman, A. Cost-effectiveness of newer technologies for the diagnosis of Mycobacterium tuberculosis infection in Brazilian people living with HIV. *Scientific reports* **10**, 21823 (2020).
6. Gomes, M. G. M. *et al.* Introducing risk inequality metrics in tuberculosis policy development. *Nat Commun* **10**, 2480 (2019).
7. Shea, K. M., Kammerer, J. S., Winston, C. A., Navin, T. R. & Horsburgh Jr, C. R. Estimated rate of reactivation of latent tuberculosis infection in the United States, overall and by population subgroup. *American journal of epidemiology* **179**, 216–225 (2014).
8. Flynn, J. L. & Chan, J. Tuberculosis: Latency and Reactivation. *Infect Immun* **69**, 4195–4201 (2001).
9. Brooks-Pollock, E. & Danon, L. Defining the population attributable fraction for infectious diseases. *International Journal of Epidemiology* **46**, 976–982 (2017).
10. Haley CA. Treatment of Latent Tuberculosis Infection. *Microbiology Spectrum*; 5 (2017).
11. Tang P, Johnston J. Treatment of Latent Tuberculosis Infection. *Current Treatment Options in Infectious Diseases* 9:4 2017; 9: 371–9 (2017).
