# Supplementary material for: Contribution of achieving poverty eradication (Sustainable Development Goal 1) to the end tuberculosis strategy in Latin America: a multi-country mathematical modelling analysis
Source: BMJ Open. 2026 Oct 27;16(10):e121015. doi: 10.1136/bmjopen-2026-121015 (PMC13723821; doi:10.1136/bmjopen-2026-121015)
Supplement: Supplementary file 2 [file bmjopen-2026-121015.reviewer_comments.pdf]

PEER REVIEW HISTORY

BMJ Open publishes all reviews undertaken for accepted manuscripts. Reviewers are asked to complete a checklist review form and are provided with free text boxes to elaborate on their assessment. These free text comments are reproduced below.

This paper was submitted to another journal from BMJ but declined for publication following peer review. The authors addressed the reviewers' comments and submitted the revised paper to BMJ Open. The paper was subsequently accepted for publication at BMJ Open.

ARTICLE DETAILS

Title (Provisional)

The Contribution of Achieving Poverty Eradication (Sustainable Development Goal 1) to The End Tuberculosis Strategy in Latin America: A Multi-Country Mathematical Modeling Analysis

Authors

Bertoni, Ana Paula Santin; Alves Rubio, Felipe Alves; Ordoñez, José Alejandro; Anderle, Rodrigo Volmir; Gestal, Priscila; Boccia, Delia; Calvacanti, Daniella; Rasella, Davide

---

VERSION 1 - REVIEW

**Reviewer**	**1**
**Name**	**Mandal, Sandip**
**Affiliation**	
**Date**	**25-Feb-2026**
**COI**	

Comments to the Author
In this article, the authors attempt to explore the relationship between poverty and tuberculosis (TB) infection, an important and relevant area of research. Although TB is often described as a disease of the poor, quantifying the precise nature and magnitude of this relationship remains challenging. Using a mathematical modelling framework, the authors seek to establish an association between poverty levels and TB incidence, and to project TB trends up to 2035 under three different poverty scenarios.

However, the methodological approach appears insufficient to adequately support their hypothesis or projections.

The most important assumption in this article concerns the risk rate (RR) for the development of active TB disease among poor populations. The authors cite the study by Bohlbro, A et al, "Active case finding of tuberculosis in general populations and at-risk groups: a systematic review and meta-analysis," published in the European Respiratory Journal (2021).

However, in that study, the "at-risk" population is defined broadly to include drug users, close contacts, the poor and marginalized, people living with HIV, and prison inmates, across different incidence strata. Thus, the reported risk rate applies to the entire composite risk group and is not estimated separately for the poor population alone. Therefore, this risk rate cannot be directly used to establish or quantify the association between poverty and TB, as it does not isolate the effect of poverty from other risk factors within the broader at-risk population.

The mathematical model employed is overly simplistic and does not capture the essential features of TB natural history and transmission dynamics. Furthermore, no calibration figure is presented. It appears that the model was calibrated using only the central estimates of WHO-reported incidence, without accounting for the associated uncertainty intervals. Ignoring uncertainty substantially limits the robustness and credibility of the projections. To capture country's program status at least they should calibrate the model with notification rate and mortality rate.

What is the distinction between new TB cases and TB incidence? It appears that the "new TB cases" reported in Table 1 correspond to TB notifications, as obtained from the WHO report.

From Table S3, it seems that the authors estimated the number of active TB cases by assuming different levels of under-notification across countries. However, since they are already using WHO-estimated incidence rates, which are adjusted for under-reporting and under-diagnosis, it is unclear why an additional assumption about under-notification is required to estimate active TB cases. The rationale for this step needs clarification.

There is also a mismatch of symbols in Figure S3, and the corresponding equations presented and described in the table. For example, the symbols "pi" and "tao" are both used for the parameter representing the rate of successful treatment, leading to inconsistency and potential confusion.

No estimated parameter values are provided, apart from the prior ranges, which themselves appear questionable.

Finally, it is not clear how the authors derived the initial conditions reported in Table S3. This aspect requires further explanation.

VERSION 1 - AUTHOR RESPONSE

Reviewer: 1

Comments to the Author

In this article, the authors attempt to explore the relationship between poverty and tuberculosis (TB) infection, an important and relevant area of research. Although TB is often described as a disease of the poor, quantifying the precise nature and magnitude of this relationship remains challenging. Using a mathematical modelling framework, the authors seek to establish an association between poverty levels and TB incidence, and to project TB trends up to 2035 under three different poverty scenarios.

However, the methodological approach appears insufficient to adequately support their hypothesis or projections.

1. The most important assumption in this article concerns the risk rate (RR) for the development of active TB disease among poor populations. The authors cite the study by Bohlbro, A et al, "Active case finding of tuberculosis in general populations and at-risk groups: a systematic review and meta-analysis," published in the European Respiratory Journal (2021). However, in that study, the "at-risk" population is defined

broadly to include drug users, close contacts, the poor and marginalized, people living with HIV, and prison inmates, across different incidence strata. Thus, the reported risk rate applies to the entire composite risk group and is not estimated separately for the poor population alone. Therefore, this risk rate cannot be directly used to establish or quantify the association between poverty and TB, as it does not isolate the effect of poverty from other risk factors within the broader at-risk population.

Answer:

We thank the reviewer for this important comment. We agree that our previous wording was not sufficiently clear and may have suggested that we used the pooled estimate for all at-risk groups from Bohlbro et al. (2021). This was not the case. In our model, we used the estimate reported specifically for the subgroup "poor and marginalised", rather than the composite at-risk group. In Bohlbro et al. (2021), this subgroup showed a yield/prevalence ratio of 3.0, compared with 1.4 in the general population, which we considered the most appropriate proxy for the excess TB risk associated with poverty. We have revised the manuscript to clarify this point in the Methods section. Specifically, we changed:

- *"The risk rate (RR) for the development of active TB disease among poor groups was derived from a published meta-analysis that systematically reviewed the prevalence of active TB in general populations and at-risk groups."*

  by

- *"The poverty-related parameter was informed by a published meta-analysis and was based specifically on the subgroup estimate for poor and marginalised populations, rather than on the pooled estimate for all at-risk groups. Bohlbro et al. (2021) reported a yield/prevalence ratio of 3.0 (95% CI: 2.0–3.9) for poor and marginalised populations, compared with 1.4 (95% CI: 0.8–1.9) in the general population."*

2. The mathematical model employed is overly simplistic and does not capture the essential features of TB natural history and transmission dynamics. Furthermore, no calibration figure is presented. It appears that the model was calibrated using only the central estimates of WHO-reported incidence, without accounting for the associated uncertainty intervals. Ignoring uncertainty substantially limits the robustness and credibility of the projections. To capture country's program status at least they should calibrate the model with notification rate and mortality rate.

Answer:

Due to the complexity of obtaining specific parameters across countries, we adopted a simpler model that could only capture the dynamics of Tuberculosis transmission to a limited extent. Therefore, we adopted the model used in Gomes et al (2019). Also, the model was calibrated using the Markov Chain Monte Carlo (MCMC) method in Python, which gives us an uncertainty

interval for the projections. Due to limited information, we opted to calibrate the new TB notifications across all countries for the period 2000 to 2019. Using the new estimated notifications, we calculated the annual incidence rate using the equation below,

Incidence rate = (New TB notified * 100,000)/(Total population)

The calibration is presented in Figure 1.

Reference: Gomes, M.G.M., Oliveira, J.F., Bertolde, A. *et al.* Introducing risk inequality metrics in tuberculosis policy development. *Nat Commun* 10, 2480 (2019). https://doi.org/10.1038/s41467-019-10447-y

3. What is the distinction between new TB cases and TB incidence? It appears that the "new TB cases" reported in Table 1 correspond to TB notifications, as obtained from the WHO report.

Answer:

We thank the reviewer for this important observation. We agree that the terminology in the previous version was not sufficiently clear and may have caused confusion. In Table 1, the variable previously labeled as "New TB cases" was based on the WHO Global Tuberculosis Programme database variable c_newinc, which corresponds to the total number of notified TB cases, including new cases, relapse cases, and cases with unknown previous treatment history. Also we clarify that "TB incidence" refers to the WHO-reported estimated incidence of tuberculosis per 100,000 population.

To address this issue, we revised the manuscript and replaced "New TB cases" with "TB notifications" in Table 1, and we reviewed every instance where the terms new TB cases and TB incidence appear and made the text clearer.

4. From Table S3, it seems that the authors estimated the number of active TB cases by assuming different levels of under-notification across countries. However, since they are already using WHO-estimated incidence rates, which are adjusted for under-reporting and under-diagnosis, it is unclear why an additional assumption about under-notification is required to estimate active TB cases. The rationale for this step needs clarification.

Answer:

Thank you for your comment. Here, we explain all of the variables and parameters shown in the Supplementary Table 3.

Table S3: Initial conditions and input variables values for each country in 2000 and between 2000-2035.

	**Brazil**	**Colombia**	**Ecuador**	**Mexico**	**Peru**
Susceptible (S0) **a**	121498031.1	34769665.38	12499705.34	88019898.7	18501714.1
Primary infection (P0) **b**	290000	40000	17500	79000	110000
Active TB (I0) (Cases by 2000 + (% of under notification)) **c**	77899 (+10%)	11630 (+15%)	6908 (+20%)	18434 (+15%)	38661 (+20%)
Latent infection (T0) **d**	$54*10^6$	4392095.12	12499705.34	9787344.20	9787344.20
Birth rate (2000-2035) **e**	3018495.78530696	760946,23674096	311613,6225938	2163015.70038792	601257,71320484
μ - Natural mortality rate (2000-2035) **f**	1/73.43	1/74.95	1/75.64	1/74.33	1/74.28

a – Susceptible (S0): For each country, the initial value of the susceptible individuals was assumed as the difference between the total population and the sum of the other compartments, that is,

$$S0=N-P0-I0-L0$$

where N is the total population in 2000.

b – Primary infection (P0): The initial value for the Primary infection was calibrated for each country based on the WHO Global Tuberculosis Programme database variable c_newinc, for the year 2000, representing the total number of notified TB cases, including new cases, relapse cases, and cases with unknown previous treatment history. The under-notification percentage was chosen based on the best model fit within a plausible range of 0% to 39.2%, informed by the WHO regional profile for the Americas, which estimated that 321,000 of 819,000 TB cases were not notified or not diagnosed in 2019 (39.2%). Source: https://www.who.int/teams/global-tuberculosis-programme/data#gtbr, and https://iris.who.int/server/api/core/bitstreams/75cba848-3a89-4e05-a736-afb69d0bece5/content

c – Active TB (I0): based on the number of new TB notifications reported in 2000 plus a country-specific percentage of under-notification, used to approximate potentially unreported cases and improve model initialization. Source: WHO Global Tuberculosis Programme database, *"Case notifications"*.

Source: https://www.who.int/teams/global-tuberculosis-programme/data#gtbr

d – Latent infection (L0): approximated as 30% of the susceptible population, based on the assumption that about 30% of individuals exposed to *Mycobacterium tuberculosis* become infected.

Source:https://bvsms.saude.gov.br/bvs/publicacoes/manual_recomendacoes_controle_tuberculose_brasil_2_ed.pdf

e - Birth rate (Λ): obtained from the World Bank Population Estimates and Projections database. For each country, the value used in the model corresponds to the average across the years included in the simulations (2000 to 2035). Source: https://databank.worldbank.org/source/population-estimates-and-projections

f – Natural mortality rate (μ): obtained from the World Bank Population Estimates and Projections database. For each country, the value used in the model is the average value of the inverse of the life expectancy across the years included in the simulations (2000 to 2035). Source: https://databank.worldbank.org/source/population-estimates-and-projections

5. There is also a mismatch of symbols in Figure S3, and the corresponding equations presented and described in the table. For example, the symbols "pi" and "tao" are both used for the parameter representing the rate of successful treatment, leading to inconsistency and potential confusion.

Answer: Thank you for the comment. We have changed the Greek letter "tau" to the symbol "pi" in the system of equations in order to match the Diagram (Figure S3) with the system.

6. No estimated parameter values are provided, apart from the prior ranges, which themselves appear questionable.

Answer:

Thank you for the comment. We included a new table in the Supplementary material (Supplementary Table S4) to show the estimated values across countries.

Table S4: Estimated parameters for the TB model.

Parameter	Country				
	**Brazil**	**Colombia**	**Ecuador**	**Mexico**	**Peru**
θ	0.730	0.730	0.730	0.730	0.730
Φ	0.1205	0.1205	0.1205	0.1205	0.1205
π	0.66	0.66	0.66	0.66	0.66
σ	0.01	0.01	0.01	0.01	0.01
μ_{TB}	0.035	0.035	0.035	0.035	0.035
β	5.510	5.550	5.510	5.550	5.550
ω	0.0001350	0.000240	0.000830	0.00020	0.000230
ν	2.5	2.6	3.5	3.8	2.8

7. **Finally, it is not clear how the authors derived the initial conditions reported in Table S3. This aspect requires further explanation.**

Answer:

We thank the reviewer for this comment. We agree that the derivation of the initial conditions was not sufficiently clear in the previous version. To address this, all initial conditions were better explained and detailed in the new **Supplementary Table S3**, as presented in our response to **Question 4**. This revised table now provides a clearer description of the country-specific initial conditions, input variables, data sources, and assumptions used to initialize the model.

VERSION 2 - REVIEW

**Reviewer**	**2**
**Name**	**Oda, Gina**
**Affiliation**	**US Department of Veterans Affairs, Public Health Surveillance and Research**
**Date**	**24-Jul-2026**
**COI**	**None**

The study is overall exceptionally clear despite the complex mathematical modelling involved. The authors provide a unique and convincing basis for understanding the effects of increasing/decreasing poverty rates on TB incidence and present a strong argument for WHO and individual governments to focus TB-reduction strategies on overall reduction in poverty levels.

Title: The title uses an abbreviation (SDG1) with which readers may be unfamiliar. I would suggest spelling out Sustainable Development Goal 1 or simply removing it from the title.

Introduction:

Well-written and provides a good basis for the study. Consider adding something about the role of poor HIV control in increasing risk for TB infection, particularly in the setting of poverty.

Page 4, line 10. I'm unclear on what the authors meant by "concentrating poverty" in the sentence. Should that be "having the highest concentration of poverty"?

Methods:

Page 3, line 40: the abbreviation "LA" is introduced here but the abbreviation has not been described previously, nor is it used later in the manuscript.

I appreciate the detailed description of statistical modelling, including supplemental figures.

Results:

Figure 1: In the description, the authors refer to an overall decreasing current trend in TB incidence, and an overall increase with the increasing poverty scenario, however, Peru's figure appears to show a somewhat flat trajectory after initial increase in the increasing poverty scenario. Do the authors have an explanation for that?

Discussion:

Page 9, lines 26-27: The authors refer to 2022 data, but I don't see data after 2019 represented in the figures or tables.

Page 9, lines 31-34: It seems like this sentence should be referring to an increase in new notifications among smaller Latin American countries, not a decrease, in the context of the previous several sentences.

Page 9, lines 34-35: Was there a decrease in incidence during 2020?

Page 9, line 48: To be clearer, the authors should not that 50% and 40% refer to the WHO targets for reduction in TB incidence (that is not clearly stated). Or, if something else is being referred to, please clarify.

Page 11, lines 5-21: Please give examples of specific CCT programs (i.e., what are the conditions?) referred to here. Also, it would be helpful to discuss the possible benefits of unconditional cash transfers or other social programs, since the focus here seems to be exclusively CCT.

Study limitations and strengths are well-described.

**Reviewer**	**3**
**Name**	**Kelson, Zoe**
**Affiliation**	**University of Exeter, Mathematics**

**Date**	**26-Aug-2026**
**COI**	**None**

Recognizing poverty's role in TB, this study forecasts the trajectory of TB incidence in the four Latin American countries with the highest TB burden under a poverty eradication Sustainable Development Goal 1 (SDG1) scenario.

Reviewer comments:

"We projected trends in new TB notifications from 2020 to 2035 using a TB compartmental model, which incorporate the poverty effects with country-specific data from 2000 to 2019. " [Abstract]

Can the modelling methodology please be further specified, albeit briefly, in the Abstract?

"The forecasted trends of our poverty eradication scenario by 2030 show reductions in a range of 32.27% (Credible Intervals [CI] 95%: 36.58%; 25.13%) to 48.56% (CI95%: 54.15%; 42.75%) in the TB incidence in comparison with the baseline, with a total number of 554,438 (CI95%: 499,713; 615,018) avoided TB notifications over the period. The increasing poverty scenario led to an incidence increase ranging from 20.02% (CI95%: 5.16%; 35.42%) to 50.20% (CI95%: 36.47%; 60.76%) in comparison with the baseline. " [Abstract]

The authors have suitably communicates uncertainty in the Abstract, Results, Tables and Figures of the article.

"The annual data on newly reported TB notifications during the years 2000 to 2019 were collected from the WHO global TB database. The poverty rate series was extracted for each country from the World Bank,12 using the threshold of 6.85 dollars per day based on the 2017 round of the International Comparison Program (ICP) price data on the International Poverty Line"

and

"Data on the general mortality rate per 1,000 people, life expectancy at birth (total in years), and total population, along with their projections up to the year 2030, were sourced from the World Bank database"

and

"Supplementary Table S2 shows the model parameters, their description and the values used in the simulations."

and

"The initial conditions and input variables for each country are detailed in Supplementary Table S3, whereas the estimated model parameters are shown in Supplementary Table S4."

The authors have suitably tabulated model inputs and parameter values, with uncertainty and source.

"In notifications where poverty values were unavailable for certain years, we interpolated the mean value between the nearest preceding and subsequent years to fill in the gaps."

Can the authors please consider undertaking a sensitivity analysis on this interpolation approach?

"To project the number of new active TB notifications from 2020 to 2035, we utilized a previously described and consolidated compartmental model, developed specifically to incorporate factors related to poverty and socioeconomic status. This model was composed of four compartments: susceptible (S), primary infection (P), latent infection (L), and active tuberculosis disease (I). The model structure is presented in Supplementary Figure S3"

and

"the transmission dynamics of TB are given by the nonlinear ordinary differential equations system (Supplementary Equation S1)"

and

"Detailed information about the model transitions diagram, the system of different equations, the parameters utilized, and the initial conditions is available on the Supplementary Material pages 4-7"

The authors suitably provide further detail on the mathematical structure of the model in the supplementary material.

"To build the scenarios, we employed an exponential decay function to reach 0% of poverty in 2030, taking into account the initial values in 2019 of each country. Also, we utilized an exponential growth function to assess a possible scenario of increasing poverty for each country. As a baseline scenario for poverty proportion, we used an AutoRegressive Integrated Moving Average (ARIMA) model. These scenarios are showed on the Supplementary Figure S4."

The authors insightfully model a range of as-if and what-if scenarios.

"We calibrated the model by utilizing the reported incidence of new tuberculosis (TB) notifications for each country, employing Markov Chain Monte Carlo (MCMC) sampling to derive posterior estimates, as implemented in the Python with the PyMC v5.7.2 package. This Bayesian methodology facilitates the fitting of the model, calibration of parameters, and estimation of the median, as well as the 5th and 95th credible intervals (CIs) for both the calibration period (2000 to 2019) and the forecasting period (2020 to 2030). We implemented four chains, each comprising 20,000 draws, to enhance the number of effective samples per iteration. To ensure the precision of the posterior analysis, we excluded the initial 2,000 samples during the burn-in phase. "

An appropriate modelling methodology has been applied by the authors.

Can the authors please consider exploring a range of one-way and probabilistic sensitivity analyses?

"Our model has limitations. First, we did not consider factors such as the potential introduction of new treatments or diagnostics, which could affect the projections. The primary aim of our study was not to explicitly forecast the future burden of TB over the next decade. Instead, we sought to understand the disparities in TB projections related to the poverty eradication goal (SDG 1) and the End TB Target strategy across four Latin American

countries. We assumed that the same factors and historical events have similarly influenced the scenarios and trends of the disease across these nations, ensuring that our comparisons remain robust in magnitude. In this context, we intentionally chose not to delve into the unique characteristics and specificities of each country, as our objective was to create a broader overview of the potential impact of poverty reduction on the burden of TB in this region. Moreover, we didn't explicitly model for COVID-19 due to the lack of quality data and the complexity of representing the healthcare disruption related to TB during this extremely challenging period. However, we assume that for the majority of analyzed countries, the situation has returned to normal in recent years and is not affecting our long-term estimates. Second, with our approach, we were only able to differentiate individuals between poor and not poor, without representing in detail more granular population characteristics. Even models that stratified compartmental models, which try to separate individuals into different groups, face challenges when it comes to representing this kind of variety.32 The only possibility to correctly account for the population's heterogeneity would be through individual-based models, which require parameters that are extremely difficult to quantify and calibrate.33 We implement a reductionist perspective,32 by addressing a multidimensional factor (poverty) in the disease dynamic, based on an innovative and recently used approach for representing SDH in infectious diseases. 8 If anything, due to these limitations mentioned earlier, our results may underestimate the true impact of poverty on TB incidence. Therefore, we should consider our findings as a lower estimate of the potential benefits of poverty eradication in these countries."

and

"The lack of data for specific subgroups in all countries limited the study to the general population by country. • This study did not consider factors such as the potential introduction of new treatments or diagnostics, due to the lack of data and complexity of the mathematical model, which could affect the projections. " [Strengths and Limitations of this Study]

Thanks to the authors for providing a copy of the STROBE checklist.

VERSION 2 - AUTHOR RESPONSE

Reviewer: 2

Comments to the Author:

The study is overall exceptionally clear despite the complex mathematical modelling involved. The authors provide a unique and convincing basis for understanding the effects of increasing/decreasing poverty rates on TB incidence and present a strong argument for WHO and individual governments to focus TB-reduction strategies on overall reduction in poverty levels.

Title: The title uses an abbreviation (SDG1) with which readers may be unfamiliar. I would suggest spelling out Sustainable Development Goal 1 or simply removing it from the title.

Answer: Thank you for the suggestion. We've changed the title to:
"The Contribution of Achieving Poverty Eradication (Sustainable Development Goal 1) to the End TB Strategy in Latin America: A Multi-Country Mathematical Modeling Analysis"

Introduction:

Well-written and provides a good basis for the study. Consider adding something about the role of poor HIV control in increasing risk for TB infection, particularly in the setting of poverty.

Answer: Thank you for this suggestion. We agree that HIV is a key driver of TB risk, particularly in the setting of poverty. We have therefore added a brief statement to the Introduction highlighting the relationship between poverty, HIV-related vulnerability, and the increased risk of progression to active TB, supported by Silva et al. (2024) and Hamada et al. (2021). Please, see page 5.

Page 4, line 10. I'm unclear on what the authors meant by "concentrating poverty" in the sentence. Should that be "having the highest concentration of poverty"?

Answer: We agree with this suggestion, and we have changed it in the manuscript. Please, see page 5.

Methods:

Page 3, line 40: the abbreviation "LA" is introduced here but the abbreviation has not been described previously, nor is it used later in the manuscript.

Answer: Thank you. We've included the abbreviation " LA " (Latin American) in the first Methods paragraph.Please, see page 6.

I appreciate the detailed description of statistical modelling, including supplemental figures.

Answer: Thank you for your kind comment and positive feedback.

Results:

Figure 1: In the description, the authors refer to an overall decreasing current trend in TB incidence, and an overall increase with the increasing poverty scenario, however, Peru's figure appears to show a somewhat flat trajectory after initial increase in the increasing poverty scenario. Do the authors have an explanation for that?

Answer: Thank you for the comment. For the Peru scenario, we attribute this to the strong declining trend in the actual incidence rate. It appears that the rise in poverty has less influence on the increase in the incidence rate than in other countries. Yet, it is sufficient to alter the strong downward trend in the incidence rate already in place before the rise in poverty.

Discussion:

Page 9, lines 26-27: The authors refer to 2022 data, but I don't see data after 2019 represented in the figures or tables.

Answer: Thank you for pointing this out. The reference to 2022 was intended to provide recent epidemiological context following the COVID-19 pandemic and was not included in the model inputs. We agree that the previous wording did not make this distinction sufficiently clear. We have therefore revised the sentence and citation accordingly. Please see page 10.

Page 9, lines 31-34: It seems like this sentence should be referring to an increase in new notifications among smaller Latin American countries, not a decrease, in the context of the previous several sentences.

Answer: Thank you for the comment. The decrease referenced in this sentence is due to the observed decrease in reported TB notifications during 2020 rather than necessarily to a true decline in TB incidence, reflecting disruptions in diagnostic services during the COVID-19 pandemic. We have revised the text to clarify this distinction and the subsequent recovery of TB diagnostic and notification services. Please see page 11.

Page 9, lines 34-35: Was there a decrease in incidence during 2020?

Answer: Thank you for this comment. The decrease observed during 2020 referred to reported TB notifications rather than necessarily to a true decline in TB incidence, reflecting disruptions in diagnostic services during the COVID-19 pandemic. We have revised the text to clarify this distinction and the subsequent recovery of TB diagnostic and notification services. Please see page 11.

Page 9, line 48: To be clearer, the authors should not that 50% and 40% refer to the WHO targets for reduction in TB incidence (that is not clearly stated). Or, if something else is being referred to, please clarify.

Answer: Thank you for this comment. We have revised the sentence to clarify that the 50% and 40% values refer to the proportion of the WHO End TB Strategy target of a 90% reduction in TB incidence by 2035. Please, see page 11.

Page 11, lines 5-21: Please give examples of specific CCT programs (i.e., what are the conditions?) referred to here. Also, it would be helpful to discuss the possible benefits of unconditional cash transfers or other social programs, since the focus here seems to be exclusively CCT.

Answer: Thank you for this suggestion. We have revised the Discussion to provide specific examples of CCT interventions, including CRESIPT in Peru and the Bolsa Família Program in Brazil, and to clarify the health- and education-related conditions commonly associated with CCTs. Please, see page 12.

Study limitations and strengths are well-described.

Answer: Thank you for your positive feedback.

Reviewer: 3

Comments to the Author:

Recognizing poverty's role in TB, this study forecasts the trajectory of TB incidence in the four Latin American countries with the highest TB burden under a poverty eradication Sustainable Development Goal 1 (SDG1) scenario.

Reviewer comments:

We projected trends in new TB notifications from 2020 to 2035 using a TB compartmental model, which incorporate the poverty effects with country-specific data from 2000 to 2019. [Abstract]

Can the modelling methodology please be further specified, albeit briefly, in the Abstract?

Answer: We revised the Abstract structure according to the journal guidelines and briefly incorporated the modelling methodology, which is a compartmental model composed of ordinary differential equations.

"The forecasted trends of our poverty eradication scenario by 2030 show reductions

in a range of 32.27% (Credible Intervals [CI] 95%: 36.58%; 25.13%) to 48.56% (CI95%: 54.15%; 42.75%) in the TB incidence in comparison with the baseline, with a total number of 554,438 (CI95%: 499,713; 615,018) avoided TB notifications over the period. The increasing poverty scenario led to an incidence increase ranging from 20.02% (CI95%: 5.16%; 35.42%) to 50.20% (CI95%: 36.47%; 60.76%) in comparison with the baseline. " [Abstract]

The authors have suitably communicates uncertainty in the Abstract, Results, Tables and Figures of the article.

Answer: Thank you for your positive feedback.

"The annual data on newly reported TB notifications during the years 2000 to 2019 were collected from the WHO global TB database. The poverty rate series was extracted for each country from the World Bank,12 using the threshold of 6.85 dollars per day based on the 2017 round of the International Comparison Program (ICP) price data on the International Poverty Line"

and

"Data on the general mortality rate per 1,000 people, life expectancy at birth (total in years), and total population, along with their projections up to the year 2030, were sourced from the World Bank database"

and

"Supplementary Table S2 shows the model parameters, their description and the values used in the simulations."

and

"The initial conditions and input variables for each country are detailed in Supplementary Table S3, whereas the estimated model parameters are shown in Supplementary Table S4."

The authors have suitably tabulated model inputs and parameter values, with uncertainty and source.

Answer: Thank you for your positive feedback.

"In notifications where poverty values were unavailable for certain years, we interpolated the mean value between the nearest preceding and subsequent years to fill in the gaps."

Can the authors please consider undertaking a sensitivity analysis on this interpolation approach?

Answer: We thank the reviewer for this suggestion. While we recognize that evaluating

interpolation assumptions through a sensitivity analysis could provide additional technical insights, this specific analysis falls outside the scope of our study. The interpolation was applied solely to bridge minor data gaps and ensure continuity in the time series, rather than serving as a central dynamic of the model.

"To project the number of new active TB notifications from 2020 to 2035, we utilized a previously described and consolidated compartmental model, developed specifically to incorporate factors related to poverty and socioeconomic status. This model was composed of four compartments: susceptible (S), primary infection (P), latent infection (L), and active tuberculosis disease (I). The model structure is presented in Supplementary Figure S3"
and
"the transmission dynamics of TB are given by the nonlinear ordinary differential equations system (Supplementary Equation S1)"
and
"Detailed information about the model transitions diagram, the system of different equations, the parameters utilized, and the initial conditions is available on the Supplementary Material pages 4-7"
The authors suitably provide further detail on the mathematical structure of the model in the supplementary material.
Answer: Thank you for your positive feedback.

"To build the scenarios, we employed an exponential decay function to reach 0% of poverty in 2030, taking into account the initial values in 2019 of each country. Also, we utilized an exponential growth function to assess a possible scenario of increasing poverty for each country. As a baseline scenario for poverty proportion, we used an AutoRegressive Integrated Moving Average (ARIMA) model. These scenarios are showed on the Supplementary Figure S4."
The authors insightfully model a range of as-if and what-if scenarios.
Thank you for your kind comment and positive feedback.

"We calibrated the model by utilizing the reported incidence of new tuberculosis (TB) notifications for each country, employing Markov Chain Monte Carlo (MCMC) sampling to derive posterior estimates, as implemented in the Python with the PyMC v5.7.2 package. This Bayesian methodology facilitates the fitting of the model, calibration of parameters, and estimation of the median, as well as the 5th

and 95th credible intervals (CIs) for both the calibration period (2000 to 2019) and the forecasting period (2020 to 2030). We implemented four chains, each comprising 20,000 draws, to enhance the number of effective samples per iteration. To ensure the precision of the posterior analysis, we excluded the initial 2,000 samples during the burn-in phase. "

An appropriate modelling methodology has been applied by the authors.

Answer: Thank you for your positive feedback.

Can the authors please consider exploring a range of one-way and probabilistic sensitivity analyses?

Answer: We thank the reviewer for this important suggestion. We would like to clarify that uncertainty in the model parameters is already explicitly incorporated through our Bayesian calibration framework. Specifically, the model parameters are estimated jointly using MCMC sampling, generating posterior distributions rather than relying on single fixed estimates. These posterior samples are then propagated through the model to obtain posterior distributions for the projected TB incidence, from which the reported posterior medians and credible intervals are derived. This procedure is described in the Methods section of the manuscript.

If, by probabilistic sensitivity analysis, the reviewer refers to evaluating how uncertainty in the model parameters propagates to uncertainty in the projected outcomes, this is already naturally incorporated into our Bayesian framework. In fact, rather than varying parameters independently or considering a limited number of predetermined values, our MCMC procedure generates joint posterior samples of all calibrated parameters. These samples represent plausible combinations of parameter values supported by both the data and the model and preserve the posterior dependence among parameters. The resulting uncertainty is then propagated simultaneously through the forecasting model.

In this sense, our Bayesian posterior analysis provides a more comprehensive characterization of parameter uncertainty than a conventional one-way sensitivity analysis, in which one parameter is varied while the remaining parameters are held fixed. A one-way analysis can be useful for identifying the isolated influence of individual parameters, but it does not account for the joint uncertainty and dependence structure among model parameters. Our approach instead evaluates the consequences of simultaneously varying the calibrated parameters according to their joint posterior distribution.

Therefore, if the reviewer's concern is primarily related to parameter uncertainty and its effect on the projected TB incidence, we believe that this aspect is already addressed by the Bayesian calibration and posterior propagation implemented in the study, and that an additional probabilistic or one-way sensitivity analysis would largely duplicate the uncertainty assessment already contained in our framework.

We have clarified this point in the revised manuscript to make more explicit how parameter uncertainty is incorporated and propagated into the reported projections. Please, see page 8.

"Our model has limitations. First, we did not consider factors such as the potential introduction of new treatments or diagnostics, which could affect the projections. The primary aim of our study was not to explicitly forecast the future burden of TB over the next decade. Instead, we sought to understand the disparities in TB projections related to the poverty eradication goal (SDG 1) and the End TB Target strategy across four Latin American countries. We assumed that the same factors and historical events have similarly influenced the scenarios and trends of the disease across these nations, ensuring that our comparisons remain robust in magnitude. In this context, we intentionally chose not to delve into the unique characteristics and specificities of each country, as our objective was to create a broader overview of the potential impact of poverty reduction on the burden of TB in this region. Moreover, we didn't explicitly model for COVID-19 due to the lack of quality data and the complexity of representing the healthcare disruption related to TB during this extremely challenging period. However, we assume that for the majority of analyzed countries, the situation has returned to normal in recent years and is not affecting our long-term estimates. Second, with our approach, we were only able to differentiate individuals between poor and not poor, without representing in detail more granular population characteristics. Even models that stratified compartmental models, which try to separate individuals into different groups, face challenges when it comes to representing this kind of variety.32 The only possibility to correctly account for the population's heterogeneity would be through individual-based models, which require parameters that are extremely difficult to quantify and calibrate.33 We implement a reductionist perspective,32 by addressing a multidimensional factor (poverty) in the disease dynamic, based on an innovative and recently used approach for representing SDH in infectious diseases. 8 If anything, due to these limitations mentioned earlier, our results may underestimate the true impact of poverty on TB incidence. Therefore, we should

consider our findings as a lower estimate of the potential benefits of poverty eradication in these countries."
and
"The lack of data for specific subgroups in all countries limited the study to the general population by country. • This study did not consider factors such as the potential introduction of new treatments or diagnostics, due to the lack of data and complexity of the mathematical model, which could affect the projections. " [Strengths and Limitations of this Study]

Thanks to the authors for providing a copy of the STROBE checklist. Answer:
Thank you for the review.
